# Supplementing linked datasets with meaningful meta-data to enable high quality research

**DOI:** 10.23889/ijpds.v1i1.300

**Published:** 2017-04-19

**Authors:** Shivani Padmanabhan, Oliver Smith, Helen Strongman

**Affiliations:** Clinical Practice Research Datalink; Health and Social Care Information Centre

## Objectives

Enable high quality research using linked data sources whose membership and coverage change over time by providing clarity in applied processing steps and meaningful meta-data.

## Approach

Our organisations have developed a process that enables linkage of primary care practice data to several disparate data sets. Identifiers are submitted to the trusted third party (TTP) organisation by consenting practices and external data controllers. These include patient NHS number, post code, date of birth and gender. The TTP remove duplicates and clean the data received, and use a sequential eight stage deterministic algorithm to match patients based on all or some of the identifiers. The TTP provide the research organisation with meta-data; a match rank variable per linked dataset to indicate at which stage in the matching algorithm the patient was matched, as well as flags to indicate whether the identifiers submitted by the practice were valid.

As part of the research organisation's standard linked data provision, only patients that have a valid NHS number in the practice data, and therefore have the potential to be linked on NHS number are identified as eligible. A flag to indicate eligibility per individual linked data source is provided. Individual data source coverage periods allow users to define follow-up time for patients. Individuals that have contributed data to more than one practice are flagged. Records for patients that have not been matched on NHS number, or who have been linked to multiple individuals in the linked dataset are removed. This together with recommendations provided in the documentation simplifies decision making for applied research. Methodological research is supported through the option to access removed records.

## Results

In the latest linkage set, identifiers for 10,272,602 patients from 404 English GP practices were collected by the TPP. Of these, 8,213,068 (80%) had a valid NHS flag. A total of 7,401,948 patients were found to have one or more records in Hospital Episodes Statistics (HES) data: 7,152,194 (97%) were matched on NHS number and 6,661,453 (93%) were identified as unique HES patients.

## Conclusion

To maximise research benefit from linked data, study designs must account for linkage methodologies and potential errors. Data providers need to support informed decision making for applied research whilst enabling methodological research that explores linkage validity and related biases. The documentation and meta-data that we provide enables users to make informed decisions about their study based on its context and design.

